# EFFECTS OF FASTING STATUS ON BLOOD-BASED INFLAMMATORY BIOMARKERS IN OLDER ADULTS

**DOI:** 10.1093/geroni/igae098.2620

**Published:** 2024-12-31

**Authors:** Christopher Engeland, Robert Tennyson, Molly Wright, Erik Knight, Seyedeh Leila Mousavi, Orfeu Buxton, Jennifer Graham-Engeland

**Affiliations:** The Pennsylvania State University, University Park, Pennsylvania, United States; The Pennsylvania State University, University Park, Pennsylvania, United States; The Pennsylvania State University, University Park, Pennsylvania, United States; University of Colorado Boulder, Boulder, Colorado, United States; The Pennsylvania State University, University Park, Pennsylvania, United States; Pennsylvania State University, University Park, Pennsylvania, United States; Pennsylvania State University, University Park, Pennsylvania, United States

## Abstract

To determine the effects of fasting status on blood-based biomarker concentrations, 30 older adults (Mage = 67.52; 70% women) fasted from midnight onwards prior to two morning laboratory visits. Each visit included 4 blood draws, spaced 1 hour apart. The visits (counterbalanced by order) were differentiated by a meal being provided after either the 2nd or 4th blood draw. This design enabled examination of fasting versus post-prandial effects, accounting for circadian variation of biomarkers. Plasma levels of cytokines (interleukin [IL]-1β, IL-4, IL-6, IL-8, IL-10, tumor necrosis factor [TNF]-α), soluble urokinase-type plasminogen activator receptor (suPAR), and C-reactive protein (CRP) were quantified at each timepoint. Separate subsets of whole blood were stimulated with 1µg/ml lipopolysaccharide and incubated for 2h or 24h at 37ºC (5% CO2) to examine cytokine responsivity to immunogenic challenge. Bayesian multilevel models (minimally informative priors, Stan MCMC sampler) compared all pre-prandial (6 timepoints) vs. post-prandial (2 timepoints) concentrations (covariates: time-of-blood-draw, visit order, age, gender, BMI). Results provided strong to very strong evidence for no changes in basal cytokines, CRP, or suPAR by fasting status. However, there was strong evidence that eating decreased stimulated TNF-α levels (2h and 24h incubation) and weak evidence that eating decreased stimulated IL-6 (2h incubation only). These findings indicate that levels of basal inflammation are not affected by eating, but that stimulated levels of TNF-α are susceptible to post-prandial effects. This implies that studies assessing stimulated TNF-α should have participants fast. Further research is needed for stimulated IL-6, which exhibited uncertain (weak) evidence of change.

